# Verifying authors’ claims to have conducted a Systematic Review? A checklist for journal editors and peer reviewers

**DOI:** 10.1186/s13750-025-00361-w

**Published:** 2025-05-14

**Authors:** Andrew S. Pullin, Biljana Macura

**Affiliations:** 1 Collaboration for Environmental Evidence, Bangor, UK; 2 School of Natural Sciences, Bangor University, Bangor, UK; 3 Collaboration for Environmental Evidence, Stockholm, Sweden; 4 Stockholm Environment Institute, Box 24218, Stockholm, 10451 Sweden

Since 2018, the Collaboration for Environmental Evidence (CEE, environmentalevidence.org) has been assessing the conduct of globally published evidence reviews relevant to environmental management [1] and collating them into the publicly available database– CEEDER (https://environmentalevidence.org/ceeder/). CEEDER is a free service to individuals or organisations who want their decision making to be informed by the best available scientific evidence. Hundreds of environmental reviews are published annually in a broad range of journals and organisations. As part of its evidence service, CEE critically appraises each review for its reliability (risk of bias), rigour, comprehensiveness, and transparency of reporting.

From data collected for CEEDER it is apparent that the large majority (over 95%) of published environmental reviews that claim to be Systematic Reviews actually fall short of the standards of conduct and reporting expected of this methodology as described by global evidence synthesis organisations such as Evidence Synthesis International, Campbell Collaboration and Cochrane, as well as CEE [2]. This misnaming of a respected methodology risks undermining evidence synthesis and the value of properly conducted Systematic Reviews to inform decision making [3]. To address this problem, CEE now provides a Checklist for Editors and Peer Reviewers (see Table 1) that covers elements of conduct and reporting that are expected in a Systematic Review.

The checklist is based on the current CEE guidance for standards of conduct (https://environmentalevidence.org/information-for-authors/) and ROSES reporting standards [4]. The checklist is designed to enable a rapid assessment of the validity of authors’ claims to have conducted a Systematic Review which implies high procedural transparency and replicability, and comprehensive, reliable and rigorous findings with minimal bias.

The checklist is structured according to the stages of Systematic Review conduct and can be used for any review or synthesis in the environmental sector that claims to provide a Systematic Review of available evidence on a specific topic. Whilst the list of questions is not exhaustive and omits many important questions that might be the subject of full peer review, it provides key questions that might quickly identify manuscripts that do not qualify as Systematic Reviews. The checklist is offered as an additional tool for editors and peer reviewers and is not intended to replace any established editorial procedures or checks a journal may have in place. ‘Yes’ to all checklist questions is expected for a Systematic Review (see guidance notes below). Where a ‘No’ is selected, editors or peer reviewers may wish to ask authors for revision of their methodology or clarification to their reporting or ultimately ask authors to withdraw their claim. The checklist will be available from the CEE website (https://environmentalevidence.org/ceeder/support-for-authors-and-editors/) and our intention is to work with the editorial community to improve the checklist using feedback from users.

We hope this checklist brings clarity for editors and peer-reviewers, assists in standardizing expectations for Systematic Reviews and ensures that authors adhere to best practices and essential methodological steps. We anticipate this checklist will be a valuable tool for improving transparency, rigor, and trust in findings of published Systematic Reviews. Finally, by increasing awareness of Systematic Review methodology and necessary requirements for review conduct and reporting, this checklist aims to enhance the reliability of environmental evidence synthesis, ultimately providing environmental policy and management with the best available evidence.

**Table 1:** A checklist for editors and peer reviewers for assessing validity of Systematic Reviews in the environmental sector

Checklist Questions	Explanatory notes	Compliance
**1. General methods**		Yes/No
Have the authors pre-registered and/or published a protocol for the review? The protocol should be cited in the review report, be freely available online and contain methodological detail of all stages of the review process	The registration/publication of an a-priori protocol for the review process to avoid post-hoc decisions that might increase risk of bias is a fundamental requirement of a Systematic Review.	
Does the review include a defined methods section providing a description of all the review stages conducted (e.g. Review question, Searching, Screening, Critical Appraisal, Data Extraction, Synthesis) in sufficient detail to enable the method to be replicated?	As with all scientific studies, the methods should be sufficiently reported so as to be replicable.	
**2. Searching for articles**		
Are all search terms and search strings, with Boolean operators (‘AND’, ‘OR’ etc.) and wildcards, clearly stated for each major source (e.g. databases, search engines) so that the exact search is replicable by a third party? Search terms for grey literature and other sources (e.g. specialist websites, search engines), if used, may be simplified.	Search strategies should be outlined in the protocol or review methods. An optimal search for literature should avoid assembling a biased or unrepresentative body of evidence and possess three key properties: comprehensive (maximises the number of potentially relevant studies found, aiming to identify all relevant studies), systematic (avoiding ad hoc search strategies reduces the susceptibility to bias resulting from e.g. no defined endpoint of search or ‘cherry picking’) and transparent (readers should be able to replicate and evaluate the search). Where possible, advice should be sought from an expert such as an information specialist/scientist.	
**3. Screening articles and including studies**		
Are study eligibility criteria precisely defined so as to be replicable (e.g. reliance on broad and potentially ambiguous terms should be avoided) and expressly related to each key element of the question? Other eligibility criteria (such as study design, publication language and date) may also be considered.	Clearly stated criteria for eligibility decisions minimise the potential for subjective decisions to influence which studies are included in the review, increase the transparency of the synthesis, and allow readers to assess the validity of the criteria to the review question. In addition to following the review question, eligibility criteria may define limits on the type of primary research to be considered in terms of (for example): geographic scope, type of data reported, type of intervention or impact, study design, date. Comprehensive searches may generate a large number of records (e.g. articles) that vary widely in their relevance to the review scope. Authors must then determine whether or not each article is sufficiently relevant (eligible) for inclusion in the data synthesis stage. However, the choice of eligibility criteria can influence the conclusions of the synthesis, and the application of inadequately defined criteria can be subjective and lead to biases. Decisions over which studies are relevant for inclusion should therefore be based on clearly defined criteria, and should be replicable and transparent.	
Is the number of records (e.g. articles) found during the searches as well as the number of records excluded at each stage of the screening process provided? For full transparency, numbers of included and included records maybe be provided via a flow diagram.	Listing all articles that were screened for eligibility and indicating whether each was included or excluded in data synthesis (usually as supplementary material), makes it clear whether potentially relevant studies have been omitted according to the eligibility criteria or were not captured by the search. Documenting the reasons for article exclusion at full text is essential for transparency and replicability.	
**4. Critical appraisal of primary study validity**		
Is an effort made to identify all sources of bias (threats to internal validity) relevant to each of the individual included studies? Ideally, authors should use a recognised critical appraisal tool.	Primary research can vary widely in methodological validity (internal validity) and study context (external validity). Internal validity can influence the findings of the research, and, if not properly accounted for, the subsequent findings of syntheses. External validity can influence the relevance/applicability of the study to users of the findings in individual contexts. Critical appraisal of internal validity (risk of bias) involves transparently evaluating the design and conduct of each included study based on methodologies, which can then help to objectively account for variation in study quality by placing greater emphasis on the most reliable studies. Online tools are available for such assessments and should be used and cited.	
**5. Data Extraction**		
Are all extracted data reported in a table or spreadsheet so that the synthesis can be replicated? This includes the data used in the synthesis from each primary study (e.g. ‘raw’ outcome metrics: means and variance measures) as well as meta-data (e.g. methodological details, population, intervention, outcomes and comparator descriptors, study context, etc.).	The volume and type of data collected by primary research articles varies substantially, even when similar questions are addressed. Authors of evidence syntheses must make decisions on which data to extract and on how to extract this information. These decisions may influence the findings of the synthesis, and so to minimise bias, the approach to data extraction should be clearly stated and, the extracted information should be comparable and consistent between studies. Transparently identifying a consistent set of data to extract from each study, for example into a structured data extraction sheet, allows the process to be replicated and evaluated by a third party, and reduces the potential for bias over which data are extracted from individual studies. Typically, extracted information from each study included in the review comprises: study aims; intervention details, study design; population characteristics; comparator details and results (point estimates and measures of variance). Providing a summary in which the population, intervention/exposure and outcome for each study are stated makes data extraction transparent, and makes it easier for readers to locate the most relevant primary literature and conduct supplementary analyses if required. Data may be provided in additional files or in an open access repository.	
**6. Data synthesis**		
Is the choice of synthesis method (i.e. quantitative meta-analysis, qualitative, or mixed-methods and narrative synthesis) described in sufficient detail to be replicable? Is the choice of the synthesis method justified on the basis of characteristics of included studies (i.e. for quantitative synthesis taking into consideration variability between studies in sample size, study design, context, outcomes, etc.)?	The approach to synthesising included studies varies substantially, and some approaches are more effective at ensuring objectivity and minimising potential bias than others. If appropriate, data should be pooled in a quantitative synthesis (e.g. meta-analysis, meta-regression). If substantial differences between populations, interventions, comparators or outcomes exist, meta-analysis (i.e. combining effect sizes across different studies) may not be appropriate. Since meta-analysis effectively treats all individual studies as part of one large study, meta-analysis is only appropriate when calculating an average effect is meaningful. If it is not appropriate to pool data across studies in meta-analysis, a reason for this should be given, and structured approach to some other quantitative or narrative synthesis taken, with efforts made to make sense of the whole of the data set, beyond describing results from individual studies in turn, noting differences in the weight of evidence behind statements made, and appropriate use of table and graphical presentations of results. Vote-counting (summing the studies which gave positive or negative findings) is not an appropriate synthesis method as an indication of impact or effectiveness.	
**7. Review limitations**		
Is an explicit section or identifiable passage of text devoted to the authors’ consideration of risk of bias in the synthesized evidence base due to limitations of the conduct of the review process as well of the primary research/data?	All reviews will have limitations, and it is important that authors are explicit about the known limitations of the primary data as well as the limitations in the methodology of the review process. Here we acknowledge the subjective nature of this criterion and the appraiser must use some subjective judgement to decide on the adequacy of any statement on limitations.	

## Electronic supplementary material

Below is the link to the electronic supplementary material.

Supplementary Material 1: A checklist for Editors and peer reviewers for assessing validity of Systematic Reviews in the environmental sectorRoberto Garbero was in charge of formating the supplement-checklist.
