# Supplementary material for: Verifying authors’ claims to have conducted a Systematic Review? A checklist for journal editors and peer reviewers
Source: Environ Evid. 2025 May 14;14:8. doi: 10.1186/s13750-025-00361-w (PMC12076875; doi:10.1186/s13750-025-00361-w)
Supplement: Supplementary file 1 — Supplementary Material 1: A checklist for Editors and peer reviewers for assessing validity of Systematic Reviews in the environmental sectorRoberto Garbero was in charge of formating the supplement-checklist. [file 13750_2025_361_MOESM1_ESM.pdf]

A checklist for Editors for assessing validity of Systematic Reviews in the Environmental sector

What is this checklist for?

Many published reviews that claim to be Systematic Reviews actually fall short of the standards of conduct and reporting expected of this methodology as described by global evidence synthesis organisations such as Evidence Synthesis International, Collaboration for Environmental Evidence (CEE), Campbell Collaboration and Cochrane Collaboration. This misnaming of a respected methodology risks undermining evidence synthesis and the value of properly conducted Systematic Reviews. To address this problem, CEE provides, with the support of BMC and the journal *Environmental Evidence*, a Checklist for Editors and Peer Reviewers of elements of conduct and reporting that are expected in a Systematic Review.

How should this checklist be used?

This checklist can be used for any review or synthesis in the environmental sector that claims to provide a Systematic Review of scientific evidence. Whilst this list is not exhaustive and omits many important questions that might be the subject of full peer review, it provides a list of key questions that can quickly identify manuscripts that do not qualify as Systematic Reviews and authors should therefore be requested to withdraw that claim. It is offered as an additional tool and is not intended to replace any established procedures or checks a journal may have in place for handling manuscripts.

'Yes' to all checklist questions are expected for a Systematic Review (see guidance notes below). Where 'No' is selected, editors and peer reviewers may wish to ask authors for revision.

About Collaboration for Environmental Evidence and CEESAT

Since 2018 the Collaboration for Environmental Evidence (CEE), an umbrella organization that sets standards and guidelines for environmental systematic evidence syntheses, is collating a database of evidence reviews, published globally, of relevance to environmental management. This is a free service to organisations with evidence needs who want their decision making to be evidence informed. Hundreds of such reviews are published annually in a broad range of journals and organisations. As part of its evidence service, CEE critically appraises each review for its reliability (risk of bias) in terms of conduct and transparency of reporting using its synthesis appraisal tool CEESAT.

Explanatory Notes

1. The Method

The production of an a-priori protocol for the review process to avoid post-hoc decisions that might increase risk of bias is a fundamental requirement of a Systematic Review. As with all scientific studies, the methods should be sufficiently reported so as to be replicable. This is equally true for reviews that aim to synthesise primary data.

2. Including Studies

Comprehensive searches may generate a large number of articles that vary widely in their relevance to the synthesis. Authors must then determine whether or not each article is sufficiently relevant (eligible) for inclusion in the data synthesis stage. However, the choice of eligibility criteria can influence the conclusions of the synthesis, and the application of inadequately defined criteria can be subjective and lead to biases. Decisions over which studies are relevant for inclusion should therefore be based on clearly defined criteria, and should be replicable and transparent.

Are eligibility criteria clearly defined?

Clearly stated criteria for eligibility decisions minimise the potential for subjective decisions to influence which studies are included in the review, increase the transparency of the synthesis, and allow readers to assess the validity of the criteria to the review question. In addition to following the review question, eligibility criteria may define limits on the type of primary research to be considered in terms of (for example): geographic scope, type of data reported, type of intervention or impact, study design, date.

Are eligibility decisions transparently reported?

Listing all articles that were screened for eligibility and indicating whether each was included or excluded in data synthesis (usually as supplementary material), makes it clear whether potentially relevant studies have been omitted according to the eligibility criteria or were not captured by the search. Documenting the reasons for article exclusion at full text is essential for transparency and replicability.

3. Critical Appraisal

Primary research can vary widely in methodological validity (internal validity) and study context (external validity). Internal validity can influence the findings of the research, and, if not properly accounted for, the subsequent findings of syntheses. External validity can influence the relevance/ applicability of the study to users of the findings in individual contexts. Critical appraisal involves transparently evaluating the design and conduct of each included study based on methodologies, which can then help to objectively account for variation in study quality by placing greater emphasis on the most reliable studies.

Does the review critically appraise each study? Documented critical appraisal, as applied to each individual study, using relevant, pre-defined critical appraisal criteria allows the author(s) of the synthesis and the reader to make more objective assessments of the relative reliability (or weighting) of each study. Some potentially relevant studies may not meet baseline methodological requirements (e.g. small sample size, pseudoreplication, spatial autocorrelation, lack of appropriate controls etc.) and so may be excluded from the synthesis. Effectively, these studies are weighted as 'zero'. Studies included in the synthesis may be treated differently according to the rigour of the sampling design, according to differences in sampling effectiveness (e.g. sample size, sampling area, study duration, etc.), or according to their generalisability for the synthesis in hand (e.g. spatial scale, study setting, etc.).

4. Data Extraction

The volume and type of data collected by primary research articles varies substantially, even when similar questions are addressed. Authors of evidence syntheses must make decisions on which data to extract and on how to extract this information. These decisions may influence the findings of the synthesis, and so to minimise bias, the approach to data extraction should be clearly stated and, wherever possible, the extracted information should be comparable and consistent between studies.

Is the method of data extraction fully documented?

Transparently identifying a consistent set of data to extract from each study, for example into a structured data extraction sheet, allows the process to be replicated and evaluated by a third party, and reduces the potential for bias over which data are extracted from individual studies. Typically, extracted information from each study included in the review comprises: study aims; intervention details, study design; population characteristics; comparator details and results (point estimates and measures of variance).

Are the extracted data reported for each study? Providing a summary in which the population, intervention/ exposure and outcome for each study are stated makes data extraction transparent, and makes it easier for readers to locate the most relevant primary literature and conduct supplementary analyses if required. Dat may be provided in additional files or in an open access repository.

5. Data Synthesis

The approach to synthesising included studies varies substantially, and some approaches are more effective at ensuring objectivity and minimising potential bias than others.

Is the choice of synthesis approach appropriate?

If appropriate, data should be pooled in a quantitative synthesis (e.g. meta-analysis, meta-regression). If substantial differences between populations, interventions, comparators or outcomes exist, meta-analysis (i.e. combining effect sizes across different studies) may not be appropriate. Since meta-analysis effectively treats all individual studies as part of one large study, meta-analysis is only appropriate when calculating an average effect is meaningful. If it is not appropriate to pool data across studies in meta-analysis, a reason for this should be given, and structured approach to some other quantitative or narrative synthesis taken, with efforts made to make sense of the whole of the data set, beyond describing results from individual studies in turn, noting differences in the weight of evidence behind statements made, and appropriate use of table and graphical presentations of results. Vote-counting (summing the studies which gave positive or negative findings) is not an appropriate synthesis method as an indication of impact or effectiveness.

6. Limitations

All reviews will have limitations and it is important that authors are explicit about the known limitations of the primary data and the conduct of the review process. Here we acknowledge the subjective nature of this criterion and the appraiser must use some subjective judgement to decide on the adequacy of any statement on limitations.
